# A Custom Mouthpiece With Lip Bumper for Osteoradionecrosis Risk Reduction After Carbon-Ion Radiation Therapy for Adenoid Cystic Carcinoma of the Lip

**DOI:** 10.1016/j.adro.2022.101114

**Published:** 2022-10-22

**Authors:** Hiroaki Ikawa, Masashi Koto, Daniel K Ebner, Hirotoshi Takiyama, Makoto Shinoto, Akihiro Nomoto, Shigeru Yamada, Hiroshi Tsuji

**Affiliations:** a QST Hospital, National Institutes for Quantum Science and Technology, Chiba, Japan; b Department of Radiation Oncology, Mayo Clinic, Rochester, Minnesota

## Introduction

The lips are a common location for minor salivary gland tumors, including adenoid cystic carcinoma (ACC).^1^ Surgery is the preferred treatment for ACC, although patients who refuse or are ineligible for surgery may receive radiation therapy (RT).^2^ However, conventional RT is ineffective in treating ACC due to inherent radioresistance. Carbon-ion radiation therapy (CIRT) has demonstrated efficacy with acceptable toxicity.^3^ Consequently, CIRT has been viewed as adaptable to treat ACC of the lip.

Osteoradionecrosis (ORN) is a serious adverse event in patients with head and neck cancer who receive CIRT. Grade ≥2 maxillary ORN after CIRT was reported at 19.0% in 2014.^4^ Further study demonstrated that the maxillary volume receiving >50 Gy (relative biological effectiveness [RBE], V_50_) CIRT delivered in 16 fractions, along with the inclusion of teeth within the planning target volume (PTV), are significant independent risk factors for ORN.^4^ To reduce the risk of maxillary ORN, a reduction of V_50_ is required, and intraoral devices are one modality by which the lip and jaw are positioned to reduce the dose. However, no devices to prevent ORN from lip cancer have been reported for CIRT.

In this technical report, we introduce and evaluate the efficacy of a custom-made mouthpiece with a lip bumper for the reduction of maxilla V_50_ in patients with ACC of the lip(s) treated with CIRT.

## Methods and Materials

### Patient

An 85-year-old male patient presented to our hospital with a primary ACC located on the right side of the upper lip without invasion of the maxilla. The patient found the esthetic impact and articulatory dysfunction after surgery to be unacceptable, and was subsequently referred for CIRT. The patient had no other known risk factors associated with maxillary ORN, including tobacco or alcohol use or a surgical history. The patient had 28 teeth, and maintained adequate oral hygiene.

### Mouthpiece

**Figure 1:**
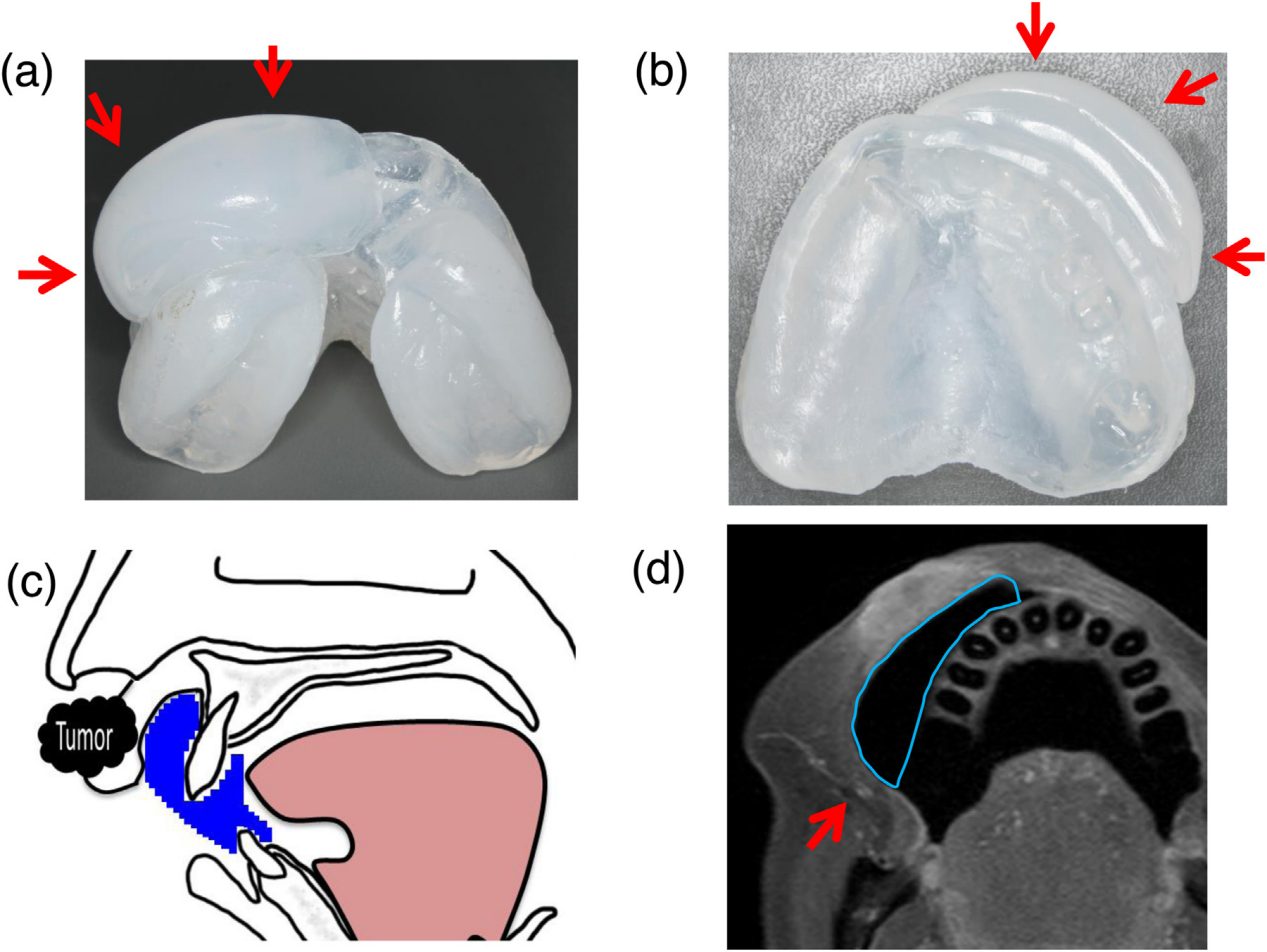
Custom-made lip bumper to protect the maxilla from irradiation. (A) Mouthpiece with lip bumper from the lingual side (arrow). (B) Mouthpiece with lip bumper from the palatal side (arrow). (C) Bumped lip position with custom-made mouthpiece. (D) T1-weighted contrast-enhanced axial magnetic resonance image of lip tumor with lip bumper.

A custom-made mouthpiece is created at our institution for all patients with head and neck cancer, as described previously.^5^^,^^6^ The mouthpiece is constructed using a thermoplastic ethylene-vinyl acetate (EVA) copolymer suitable for use in charged-particle therapy.^7^ For lip tumors, the maxilla is incorporated into the PTV because of its anatomic adjacency to the usual lip position. Therefore, a bumper moves the lip anteriorly to protect the maxilla from irradiation (Fig. 1A, 1B). Lip bumpers are orthodontic devices that help create a larger space between the front of the teeth and the lip.^8^^,^^9^ For the lip bumper in this case, the EVA base occupied the entire oral vestibule, excluding the superior labial frenulum (Fig. 1C, 1D).

### CIRT

**Figure 2:**
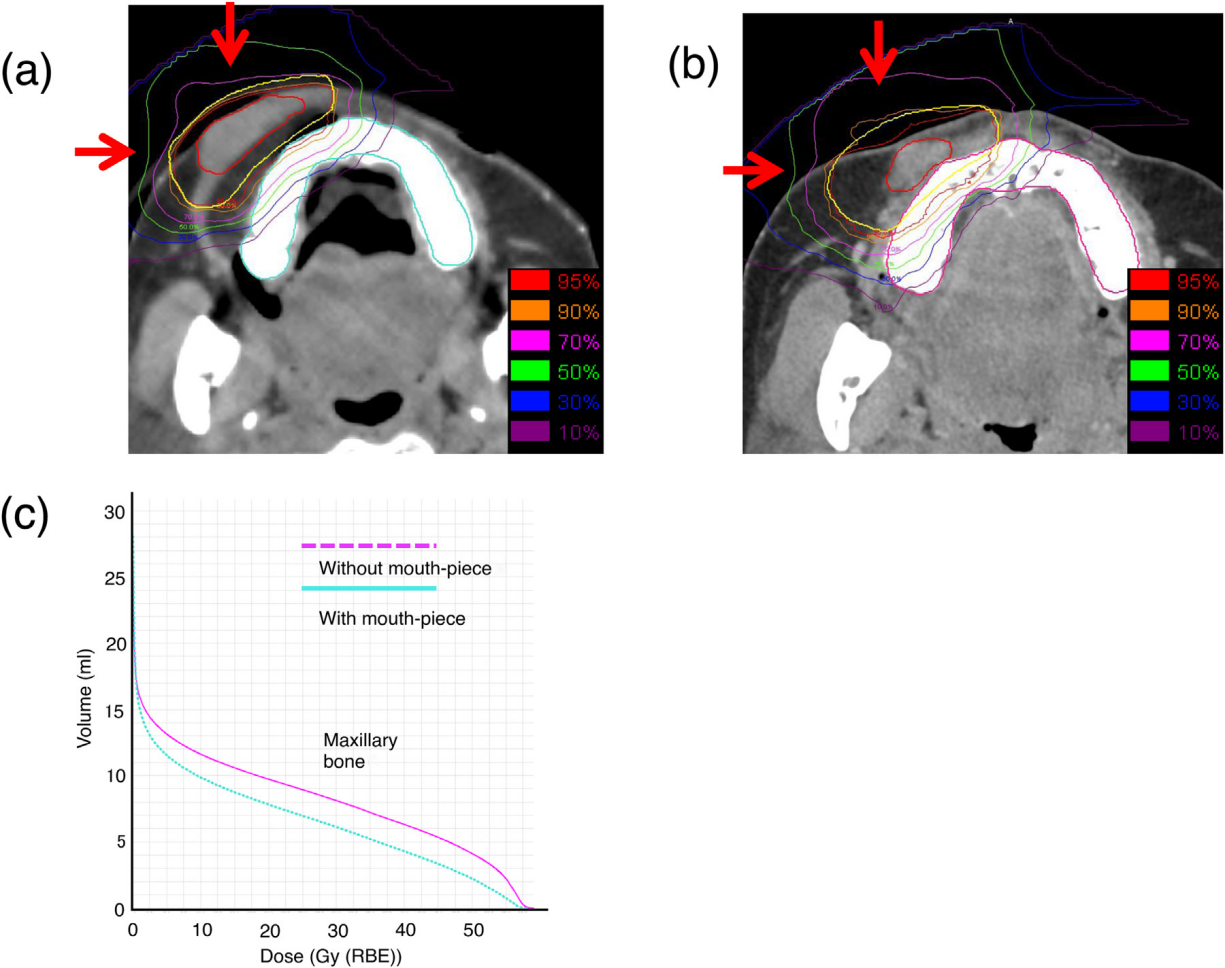
Dose distribution in the maxilla. (A) Actual clinical results of dose distribution with the mouthpiece. (B) Clinical results of dose distribution without the mouthpiece in a simulated treatment. (C) Dose-volume histogram for maxilla with and without the mouthpiece. Contours in red (gross tumor volume) and yellow (planning target volume).

The CIRT technique has been described previously.^10^ After positioning and replicable immobilization, the gross tumor volume was defined on the fusion of magnetic resonance imaging with 2-mm computed tomography (CT) imaging, with the clinical target volume including a 5- to 7-mm expansion of the gross tumor volume. The PTV was generated with an additional 2- to 3-mm margin from the clinical target volume. In this case, 57.6 Gy (RBE) CIRT was delivered in 16 fractions over 4 weeks, with 4 treatments per week in line with institutional scheduling. Treatment planning was performed using Xio-N (ELEKTA, Stockholm, Sweden; Mitsubishi Electric, Tokyo, Japan). The dose distribution and port angles are shown in Fig. 2A. Two ports were used, and scanning CIRT was employed.

### Follow-up and ORN evaluation

After CIRT, maxillary ORN was evaluated based on clinical symptoms, macroscopic examination, and the findings of the CT and magnetic resonance imaging conducted every 2 to 3 months. The National Cancer Institute's Common Terminology Criteria for Adverse Events, version 3.0, was used to define toxicity.

### Simulation study

A simulation study was conducted to evaluate spacer utility. Maxillary V_50_ with and without the spacer was calculated. A dose-volume histogram (DVH) analysis was performed using MIM software (MIM Software Inc, Cleveland, OH) to compare the irradiation dose with the maxilla volume with and without the mouthpiece. Pretreatment diagnostic CT images in the same case were used to simulate treatment without a mouthpiece (Fig. 2B). The maxillary DVH included both the alveolar and palatine processes of the maxilla, but the maxillary sinuses were excluded.

## Results

CIRT was completed over a period of 29 days as scheduled. During CIRT, grade 1 dermatitis and grade 3 mucositis appeared in the upper lip and labial gingiva, respectively, but no mucositis was observed in the palatal mucosa. The mouthpiece itself elicited pain during treatment due to its direct contact with the oral mucosa, which was controlled with nonsteroidal anti-inflammatory drugs and 2% lignocaine gel. During the treatment, there was no change in the gingiva, but the upper lip swelled slightly; however, the swelling was not significant enough to affect the accuracy of the treatment.

**Figure 3:**
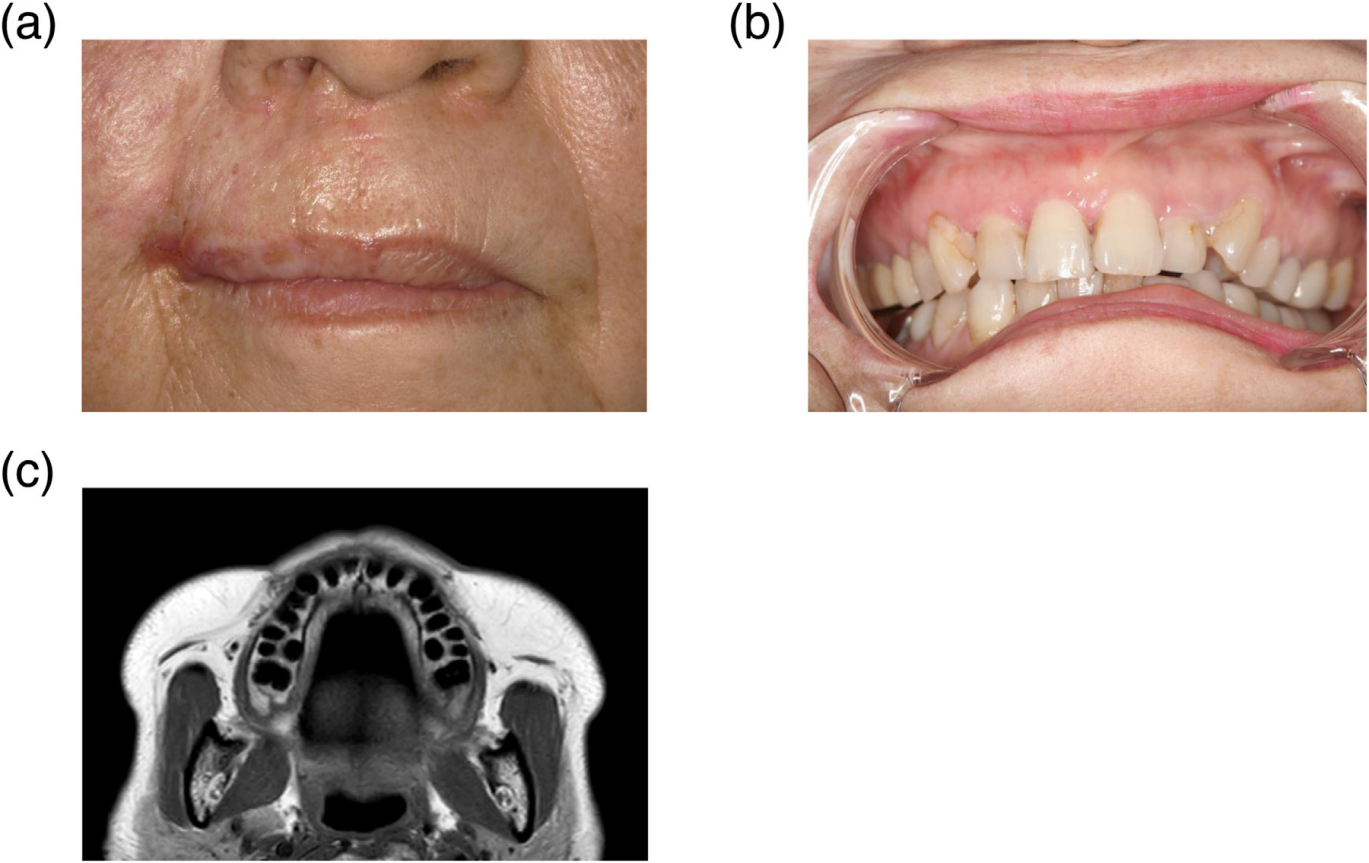
Clinical results 8 years after carbon-ion radiation therapy. (A) Skin findings. (B) Oral findings. (C) Maxillary bone findings on T1-weighed axial magnetic resonance images.

Eight years after CIRT, no late dermatitis (Fig. 3A), oral mucositis, dental issues (eg, dental caries, periapical abscesses, and periodontitis; Fig. 3B), or ORN was observed (Fig. 3C), and lip ACC was controlled clinically and radiologically.

In the simulated treatment without the mouthpiece, the maxilla was irradiated with a high dose (Fig. 2B). With mouthpiece usage, the upper lip was extended and the tumor laterally enlarged compared with nonusage. In the comparison study, the DVH analysis demonstrated a reduction in dose in the high-dose area of the maxilla with mouthpiece usage (V_50_ = 2.21 mL) compared with nonusage (V_50_ = 4.11 mL; Fig. 2C). In the PTV, 1 and 4 teeth were present when the mouthpiece was and was not used, respectively.

## Discussion

In limited cases, CIRT may be indicated for head and neck nonsquamous cell carcinoma (SCC).^3^ In patients with lip ACC who find the esthetic change and articulatory dysfunction caused by resection unacceptable, CIRT appears to be a potential alternative for definitive treatment. Therefore, upon consideration of ORN risk, which may ultimately lead to surgical intervention, careful deployment of dose volumes while maximally sparing the maxilla is required. Herein, an ACC of the lip in close proximity to the maxilla was treated with CIRT using a custom-fitted mouthpiece to reduce the maxillary dose.

A previous study reported on the independent risk factors to develop maxillary ORN after CIRT. On multivariate analysis, V_50_ (≥3.0 mL) and the presence of teeth in the PTV were identified as independent risk factors.^4^ Consequently, efforts to reduce the volume of the maxilla receiving a radiation dose of >50 Gy (RBE) have been ongoing. Herein, the DVH analysis revealed a reduction in V_50_ from 4.11 mL to 2.21 mL with a mouthpiece. The lip bumper appears effective in reducing maxillary dose and, by extension, the risk of maxillary ORN for lip ACC treated with CIRT.

Similarly, dosimetric parameters have been reported as risk factors for ORN in the mandible. Musha et al reported that doses of 30 Gy (RBE) to the mandible and teeth caused ORN at 29.5 ± 6.7 cc and 3.9 ± 1.8 cc, respectively, with cutoff values of 16.5 cc and 1.8 cc, respectively.^11^ The mouthpiece described may be applied to lower lip tumors as well, which may reduce the risk of mandibular ORN.

Lip cancer is a common cancer of the head and neck, and is generally SCC.^12^ Definitive treatment of early lip SCC involves surgical resection or RT, generally incorporating either conventional photon RT or brachytherapy.^2^ Cases of lip SCC treated with RT show good disease control,^13^ but there is a risk of ORN.^14^ Spacer usage has been described in the brachytherapy literature for lip SCC,^15^^,^^16^ but there have been no such reports on photon RT to date. For lip cancer with no extension to the jawbone, photon RT using a lip bumper may be effective, sparing the maxilla from irradiation and preventing the risk of ORN.

In non-SCC cases requiring escalated treatment doses, charged particle therapy (eg, CIRT and proton beam therapy [PBT]) may be considered, offering improvement in RBE and dose distribution in comparison with conventional RT. Charged particle therapy, in particular, should be considered for radioresistant diseases.^17^ To date, only one report has been published on the use of a spacer using the lip bumper technique for lip tumors in PBT.^18^ Srivastava et al reported on the fabrication and benefits of a modified radiation stent made of heat-polymerized acrylic resin with bilabial protrusion of the lips, which was used in PBT for salivary polymorphous adenocarcinoma of the upper lip.^18^ However, their clinical report did not evaluate the radiation dose to the maxilla. In our simulation study, we assessed the dose to the maxilla with and without the mouthpiece with a lip bumper and demonstrated the usefulness of the lip bumper.

At our hospital, we use floor-mounted kV x-ray image guided RT systems^19^ for patients with head and neck cancer during CIRT. The mouthpiece material, EVA, has a low CT number of approximately –66.85 Hounsfield units,^7^ and is not clearly visible on x-ray images. However, the correct fit of the mouthpiece in the oral cavity can be recognized by x-ray images of the maxilla and mandible positions. The position of the mouthpiece and lips is also confirmed by marking the position of the upper lip with a line on the mouthpiece and directly observing each irradiation.

## Conclusion

A custom-made mouthpiece with a lip bumper was employed in CIRT to reduce the maxillary dose. The patient tolerated treatment well with acceptable treatment toxicity, and the DVH simulation demonstrated a 46% reduction in maxillary dose with expected concomitant reduced ORN risk. Further evaluation in a dedicated cohort is warranted. In addition, evaluation of the dental sequelae of CIRT is also a future concern.

## Acknowledgments

The authors thank Minami Ikawa for the preparation of the medical illustrations.
